# Short-Term Non-Decaying Mechanoluminescence in Li_2_MgGeO_4_:Mn^2+^

**DOI:** 10.3390/ma13061410

**Published:** 2020-03-20

**Authors:** Yi-Fei Zhu, Tong Jiang, Lei Li, Long-Xiang Cheng, Jun-Cheng Zhang

**Affiliations:** College of Physics, Qingdao University, Qingdao 266071, China; zhuyifei1994@163.com (Y.-F.Z.); jiangtong034@163.com (T.J.); 15769062880@163.com (L.L.); chenglx12138@163.com (L.-X.C.)

**Keywords:** mechanoluminescence, traps, phosphors

## Abstract

Trap-controlled mechanoluminescent (ML) materials characterized by reproducible mechanoluminescence (ML) after irradiation recharging have shown attractive prospects in applications including stress distribution visualization, stress-driven light sources, and anti-counterfeiting. However, these materials generally suffer from the difficulty of achieving non-decaying ML when subjected to continuous mechanical stimulation. Herein, we develop a trap-controlled reproducible ML material, Li_2_MgGeO_4_:Mn^2+^, and report its short-term non-decaying ML behavior. Investigation of trap properties suggests that the unique non-decaying ML behavior should arise from the deep traps existing in Li_2_MgGeO_4_:Mn^2+^, which provide electron replenishment for shallow traps that release small numbers of electrons during short-term cyclic friction. Our results are expected to provide a reference for the ultimate achievement of long-term non-decaying ML in such materials.

## 1. Introduction

Mechanoluminescent (ML) materials that can quantitatively convert mechanical stimuli into light emission have drawn great attention due to potential applications ranging from stress distribution visualization [1,2] and structural health diagnosis [3,4,5] to light sources [6,7,8,9,10,11,12,13] and anti-counterfeiting [14,15,16]. Dozens of inorganic ML materials with reproducible mechanoluminescence (ML) have been developed during the past two decades [17,18]. Based on whether ML requires light irradiation to recover, the reproducible ML materials can be divided into two types. One type that does not require irradiation is called self-reproducible ML materials [18]. For example, ZnS:Mn^2+^ and ZnS:Cu^+^ exhibited robust and non-decaying ML over many thousands of cycles of a mechanical stimulus [1,19,20]. Such long-term non-decaying ML behavior evidently facilitates quantitatively visualizing and detecting continuously applied stress. However, self-reproducible ML materials are mainly limited to transition metal-doped ZnS series and the underlying mechanism of self-reproducibility remains unclear. Another type that requires irradiation is trap-controlled ML materials [18]. Most developed reproducible ML materials are of this type, such as SrAl_2_O_4_:Eu^2+^,Dy^3+^ [21], CaYAl_3_O_7_:Ce^3+^ [22], BaSi_2_O_2_N_2_:Eu^2+^ [23], Sr_3_Sn_2_O_7_:Sm^3+^ [24], and LiNbO_3_:Pr^3+^ [25], as well as CaZr(PO_4_)_2_:Eu^2+^ [26], (Ca,Sr)_2_Nb_2_O_7_:Pr^3+^ [27,28], Ca_3_Ti_2_O_7_:Pr^3+^ [29], NaNbO_3_:Pr^3+^,Er^3+^ [14], and La_1.95_Ti_2_O_7_:Pr^3+^ [15], developed by our lab. These materials employ carrier traps to store excitation energy from light irradiation (e.g., ultraviolet (UV) excitation). Under continuous mechanical stimuli, the stored energy is released gradually, resulting in a decayed ML. The next irradiation would recharge the emptied traps and recover the decayed ML; nevertheless, it complicates the operation process in diverse potential applications. In order to overcome this decaying behavior, Kim et al. introduced a continuous-irradiation technology to consistently recharge the emptied traps during the cyclic loading process [30]. However, the continuous UV radiation simultaneously excites photoluminescence of phosphors, which reduces the signal-to-noise ratio of ML. Therefore, it is still a significant challenge to realize non-decaying ML in trap-controlled reproducible ML materials from the aspects of not only proposing feasible solutions but also advancing practical applications.

Herein, we develop a trap-controlled reproducible ML material, Li_2_MgGeO_4_:Mn^2+,^ and report its short-term non-decaying ML behavior. Li_2_MgGeO_4_:Mn^2+^ belongs to the group of green-emitting long-persistent phosphors [31,32,33]; however, there has never been a report on the phenomenon related to its ML, let alone the idiographic ML behavior discovered in this work. Through the investigation of ML and trap properties, we attribute its short-term non-decaying ML to the electron replenishment from deep traps to shallow traps. We finally propose a possible mechanism to interpret the unique ML process in Li_2_MgGeO_4_:Mn^2+^.

## 2. Materials and Methods

### 2.1. Synthesis

A series of Li_2_Mg_1-x_GeO_4_:xMn^2+^ (x = 0, 0.25, 0.5, 0.75 and 1 mol.%) materials was synthesized by high-temperature solid-state reaction among Li_2_CO_3_ (99.99%, Aladdin, Shanghai, China), 4MgCO_3_·Mg(OH)_2_·5H_2_O (99.99%, Aladdin, Shanghai, China), GeO_2_ (99.99%, Aladdin, Shanghai, China), MnCO_3_ (99.95%, Aladdin, Shanghai, China); if not specified, the Mn^2+^ concentration is fixed at 0.5 mol.%. Stoichiometric raw materials ground thoroughly were firstly pre-calcined at 900 °C for 4 h in air. The pre-calcined materials were then ground again and pressed into disks (10 mm in diameter and 1 mm thick) at 20 MPa. Subsequently, the disks were sintered in an alumina crucible at 1200 °C for 5 h in air and naturally cooled. Parts of the sintered ceramic disks were ground and screened through a 20 μm sieve to produce microparticles. In order to characterize their ML properties, composite disks (25 mm in diameter and 15 mm in thickness) were prepared by embedding the screened particles in optical epoxy resin (SpeciFix, Struers, Shanghai, China) at a weight ratio of 1:9. 

### 2.2. Characterization

Phase structure was investigated by powder X-ray diffraction (XRD, D8 Advance, BRUKER AXS GMBH, Karlsruhe, Germany). Observations of high-resolution transmission electron microscopy (HRTEM) were carried out on a JEOL JEM-2100F (JEOL, Tokyo, Japan) microscope. The diffuse reflectance spectra were measured using a ultraviolet/visible/near infrared spectrophotometer (V570, Jasco, Tokyo, Japan). Afterglow (AG) curves were characterized by a fluorescence spectrometer (F-4600, Hitachi, Tokyo, Japan). Thermoluminescence (ThL) was measured using a ThL meter (FJ427A1, Beijing Nuclear Instrument Factory, Beijing, China) at a heating rate of 1 °C/s. Compression and friction were produced by a universal testing machine and a lab-made friction machine, respectively. ML signals were captured using an in-house assembled photon-counting system. Spectra of ML and AG were recorded by an optic spectrometer (QE65000, Ocean Optics, Florida, United States). Before the measurement of ML and AG, the samples were irradiated by a handheld UV lamp (254 nm, 6 W) for 1 min. Photographs of ML and AG were recorded using a camera (EOS 7D Mark II, Canon, Tokyo, Japan). All measurements except AG were performed at room temperature.

## 3. Results and Discussion

**Figure 1:**
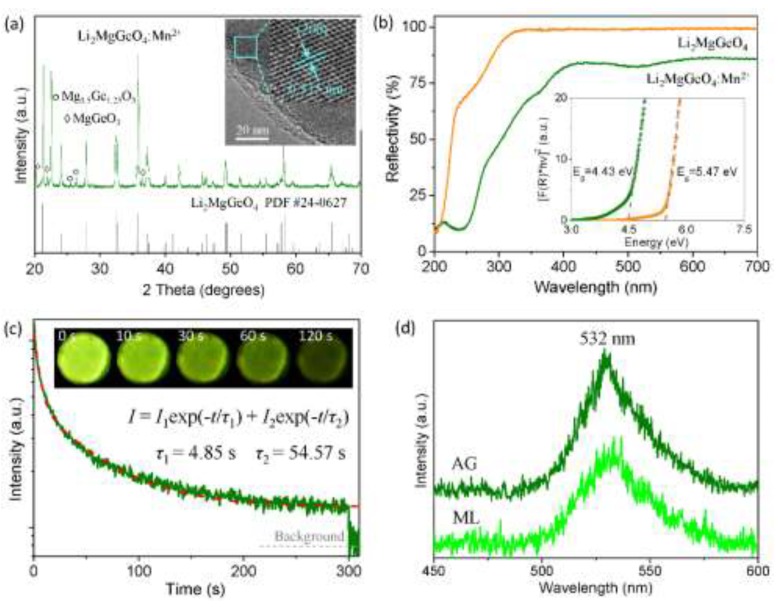
(**a**) XRD pattern of Li_2_MgGeO_4_:Mn^2+^ powders. The inset shows the high-resolution transmission electron microscopy (HRTEM) image of an individual particle. (**b**) Diffuse reflection spectra of Li_2_MgGeO_4_ and Li_2_MgGeO_4_:Mn^2+^ powders. The inset plots the derived optical bandgap. (**c**) Afterglow (AG) curve of Li_2_MgGeO_4_:Mn^2+^ by monitoring the emission wavelength of 532 nm. The inset presents the optical images taken at different delay times (0–120 s) after UV irradiation for 1 min. (**d**) AG and mechanoluminescence (ML) spectra of Li_2_MgGeO_4_:Mn^2+^.

The XRD pattern of the as-synthesized materials shows a main phase of Li_2_MgGeO_4_ combined with a small amount of impurities, which possibly arise from lithium volatilization in high-temperature sintering (Figure 1a). The Li_2_MgGeO_4_ host has an orthorhombic structure with space group *Pmn*2_1_, which provides a non-centrosymmetric lattice to generate a piezoelectric response under external pressure [34,35]. The HRTEM image of an individual Li_2_MgGeO_4_:Mn^2+^ particle demonstrates a *d*-spacing of 0.315 nm corresponding to the facet (200) of orthorhombic Li_2_MgGeO_4_ (inset of Figure 1a). The doped Mn^2+^ ions are considered to enter the Mg^2+^ sites due to the same valence state and the similar radii of Mg^2+^ (0.57 Å, CN = 4) and Mn^2+^ (0.66 Å, CN = 4) [36]. Measurement of the diffuse reflection spectra, and analysis based on the Kubelka–Munk function [37] and the Tauc relation [38], indicate that Mn^2+^ doping causes a decrease of the optical band gap from 5.47 eV of Li_2_MgGeO_4_ to 4.43 eV of Li_2_MgGeO_4_:Mn^2+^ (Figure 1b). The decrease in reflectivity after Mn^2+^ doping should result from the absorption of Mn^2+^ and the formation of carrier traps. Furthermore, Mn^2+^ doping enables Li_2_MgGeO_4_:Mn^2+^ to show a property of persistent luminescence. For example, after exposure of the as-synthesized Li_2_MgGeO_4_:Mn^2+^ material to 254 nm light irradiation for 1 min, we can observe a green AG with the naked eye for ~3 min (Figure 1c). The AG curve measured by monitoring the emission wavelength of 532 nm of Li_2_MgGeO_4_:Mn^2+^ can be well fitted with a bi-exponential decay equation, revealing a rapid decay process with τ_1_ = 4.85 s and a slow decay process with τ_2_ = 54.57 s. The results imply the formation of traps with different depths (shallow and deep traps) in Li_2_MgGeO_4_:Mn^2+^. Characterization of emission spectra of AG shows that the AG of Li_2_MgGeO_4_:Mn^2+^ originates from the ^4^T_1_(^4^G)–^6^A_1_(^6^S) transition of Mn^2+^ coordinated tetrahedrally (Figure 1d) [31]. These trap levels may come from defects generated during the process of material preparation, including lithium vacancies, oxygen vacancies, and doping traps [18].

**Figure 2:**
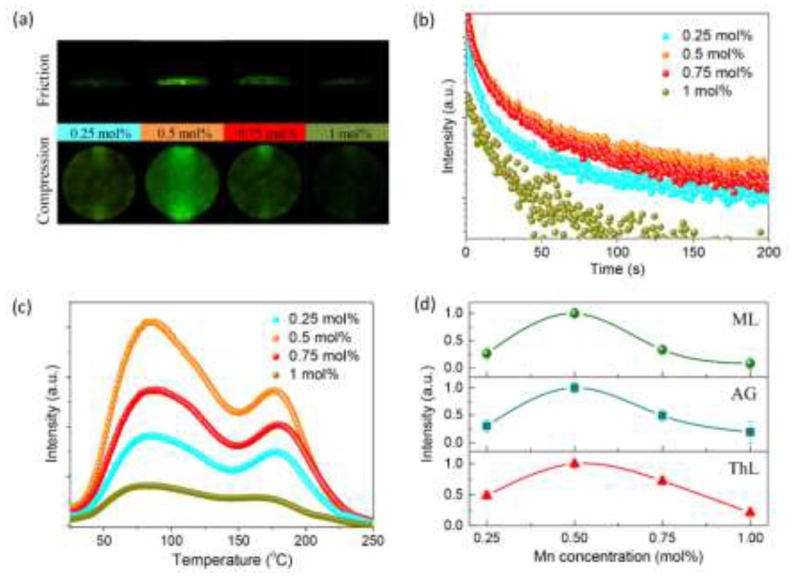
Characterization of ML, AG, and thermoluminescence (ThL) in Li_2_MgGeO_4_:Mn^2+^ with different Mn^2+^ concentrations. (**a**) Optical images of ML induced by friction (upper) and compression (bottom). (**b**) AG curves. (**d**) ThL curves. (**c**) Dependence of ML intensity, AG intensity, and integrated ThL intensity on Mn^2+^ concentration.

ML characterization demonstrates that Li_2_MgGeO_4_:Mn^2+^ can respond to non-destructive mechanical stimuli including friction and compression by ML (Figure 2a). The ML spectrum is consistent with the AG spectrum, indicating that ML in Li_2_MgGeO_4_:Mn^2+^ is also caused by the ^4^T_1_(^4^G)–^6^A_1_(^6^S) transition of Mn^2+^. Investigations of the ML, AG, and ThL properties of Li_2_MgGeO_4_:Mn^2+^ with different Mn^2+^ concentrations reveal a similar trend in the variation of the ML, AG, and ThL intensity as Mn^2+^ concentration increases (Figure 2b–d). These results suggest the ML in Li_2_MgGeO_4_:Mn^2+^ might arise from trap levels that are homologous to AG. 

**Figure 3:**
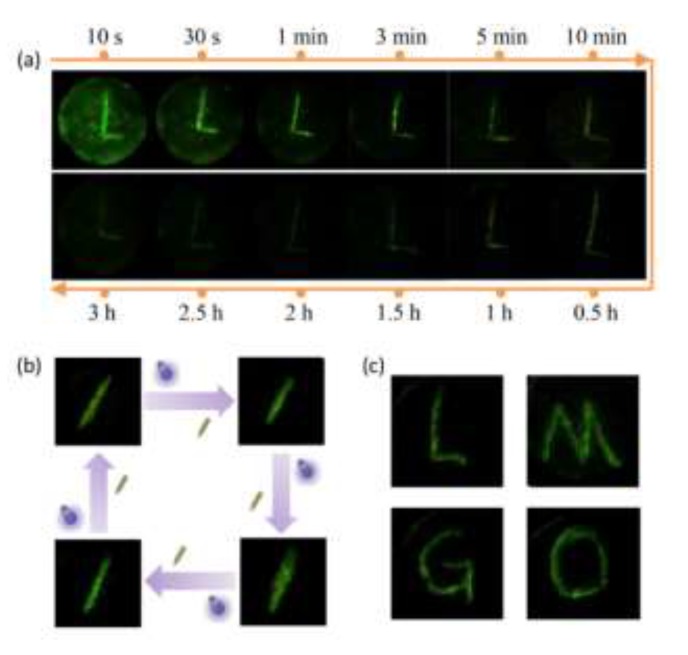
Optical photographs of ML induced by handwriting in diverse conditions. (**a**) Visualization of ML generated by handwriting letter “L” at different delay times (10 s–3 h). (**b**) Recoverable ML after UV irradiation. (**c**) ML generated by handwriting different letters on the same sample after UV irradiation.

The ML of Li_2_MgGeO_4_:Mn^2+^ exhibits typical features of trap-controlled reproducible ML materials; that is, long-term decay behavior and reproducibility after irradiation. For example, after performing 254 nm light irradiation for 1 min, the letter “L” was written on an irradiated composite cylinder after different delay times (10 s–3 h). We can clearly observe that as the delay time increases, the ML intensity of handwriting gradually becomes weaker due to the de-trapping of trapped charge carriers. Nevertheless, it should be noted that the ML of Li_2_MgGeO_4_:Mn^2+^ is still clearly visible to the naked eye within three hours (Figure 3a). The AG intensity also gradually weakens, but the decay rate is faster (visible to the naked eye for ~3 min), indicating that the ML trap is much deeper than the AG trap, as reported in other ML materials [14,18,23,28]. The attenuated ML can be recovered after light recharging of UV irradiation (Figure 3b), indicating the excellent reproducibility of ML in Li_2_MgGeO_4_:Mn^2+^. By combining the characteristics of ML display and reproducibility, we can write arbitrary letters on the same sample, such as the letters “L”, “M”, “G”, and “O”, representing the host lattice of Li_2_MgGeO_4_:Mn^2+^ (Figure 3c).

**Figure 4:**
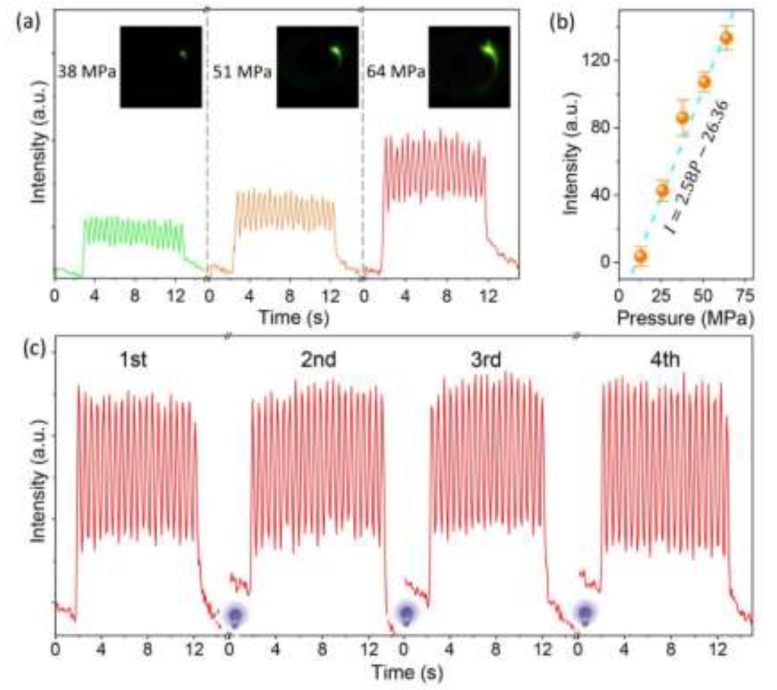
(**a**) Friction-induced ML curves under different pressures, showing a stable ML response during ~20 cycles (4π rad/s). The insets show the instantaneous ML photographs. (**b**) Linear relation between ML intensity and pressure. (**c**) Repeatability and stability test under cyclic friction (64 MPa, 4π rad/s) and UV irradiation (254 nm, 1 min).

The most attractive ML feature of Li_2_MgGeO_4_:Mn^2+^ is its short-term non-decaying ML behavior, which is quite distinct from the reported trap-controlled reproducible ML materials characterized by the decaying behavior of ML [14,15,18,21,23,26,27,28,29]. During an experiment using continuous rod friction (4π rad/s) at different pressures, the ML signal showed a nearly stable periodic oscillation during ~20 cycles (Figure 4a). When the pressure was increased from 13 to 64 MPa, the ML intensity gradually improved and showed a linear dependence on the applied pressure (Figure 4b), which is extremely useful for visual observation of stress distribution. However, it is worth noting that the ML always maintains the short-term non-decaying characteristic at different pressures (~20 cycles of mechanical friction during ~10 s), and the signals of ML demonstrate an excellent signal-to-noise ratio throughout. Furthermore, although ML is still attenuated under long-term cyclic friction, short-term non-decaying ML behavior shows stable reproducibility after UV irradiation (Figure 4c). These results indicate that carrier traps might play an important role in the short-term non-decaying ML of Li_2_MgGeO_4_:Mn^2+^.

**Figure 5:**
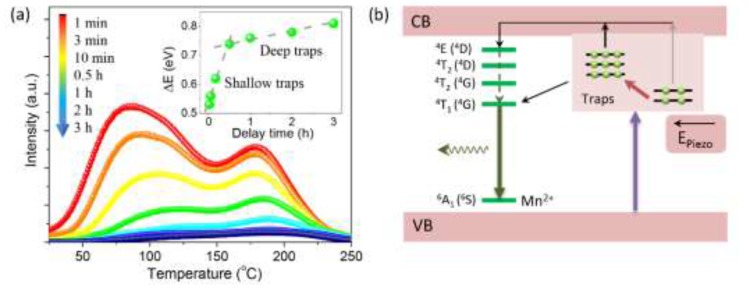
(**a**) ThL curves measured at different delay times. The inset plots the derived trap depth versus the delay time. (**b**) Schematic illustration of the proposed ML process, involving electron replenishment from deep traps to shallow traps.

In order to interpret the underlying mechanism possibly related to the trap levels, we measured the ThL curves of Li_2_MgGeO_4_:Mn^2+^ subjected to different delay times after UV irradiation (Figure 5a). The ThL curves consist of two main peaks respectively located in low-temperature (25–150 °C) and high-temperature (150–250 °C) regions, suggesting the co-existence of shallow and deep traps. By using the initial rise method [39], the trap depth was evaluated, i.e., 0.53–0.74 eV for shallow traps and 0.74–0.81 eV for deep traps (inset of Figure 5a). It should be further noted that both the low-temperature and high-temperature peaks are weakened with the increase of the delay time, which indicates that the shallow and deep traps are simultaneously de-trapping carriers to participate in light emission. Considering the given number of trapped carriers after UV irradiation, we infer that it possibly occurs carrier replenishment from deep traps to shallow traps during the ML process, thereby supporting the unique short-term non-decaying ML behavior in Li_2_MgGeO_4_:Mn^2+^. This inference can be supported by the reported ThL and ML results of other trap-controlled reproducible ML materials. For example, NaNbO_3_:Pr^3+^ [14], La_1.95_Ti_2_O_7_:Pr^3+^ [15], and Ca_3_Ti_2_O_7_:Pr^3+^ [29] only have ThL peaks below 150 °C (corresponding to shallow traps), while there are no high-temperature ThL peaks (corresponding to deep traps, such as the 150–250 °C ThL peak in Li_2_MgGeO_4_:Mn^2+^). On the other hand, all of these materials show a typical decaying behavior even under short-term cyclic frictional stimuli [14,15,29]. Therefore, we reasonably ascribed their continuous decaying behavior to the gradual emptying of shallow traps and the absence of carrier replenishment from deep traps.

According to the above results and the piezoelectricity-induced ML model [18,19,20,21,22,23,24,25,26,27,28,29,40], we describe the possible ML mechanism in Li_2_MgGeO_4_:Mn^2+^ as follows (Figure 5b). Under UV irradiation, the excited electrons of Mn^2+^ ions are captured by electron traps, which localize near the conduction band (CB). Both shallow and deep traps would be filled, and deep traps may be filled from shallow traps via non-radiative relaxation. Due to the need for higher activation energy to de-trap, the electrons captured by deep traps have a longer lifetime than electrons captured by shallow traps at ambient temperature. When mechanical stimuli are applied to generate a piezoelectric field on the crystal lattice, the trapped electrons in shallow traps become easier to de-trap and return to the excited Mn^2+^ ions (through CB transport and/or the tunneling effect) to produce ML via the ^4^T_1_(^4^G)–^6^A_1_(^6^S) transition. Once a small number of trapped electrons are released from the shallow traps, the deep traps can replenish electrons to the shallow traps, which supports the non-decaying output of ML in the short term. Considering that only a limited number of electrons are captured by the traps in one irradiation, the ML intensity gradually decreases with the release of the captured electrons for a long time. However, the next irradiation will recover the ML intensity by filling the emptied traps.

## 4. Conclusions

We have reported an idiographic short-term non-decaying ML behavior in the developed Li_2_MgGeO_4_:Mn^2+^. On the one hand, similar to traditional trap-controlled reproducible ML materials, the ML of Li_2_MgGeO_4_:Mn^2+^ shows typical long-term decaying behavior (up to 3 h) and reproducibility after irradiation (254 nm for 1 min), which are derived from the emptying and refilling of the carrier traps, respectively. On the other hand, in contrast to traditional ML materials, the ML of Li_2_MgGeO_4_:Mn^2+^ features a short-term non-decaying behavior under cyclic mechanical friction with different loads (~20 cycles in ~10 s, 13–64 MPa). Investigation of trap properties indicates the existence of shallow traps (0.53–0.74) and deep traps (0.74–0.81 eV) in Li_2_MgGeO_4_:Mn^2+^. Structural analysis reveals that the piezoelectric effect arising from the non-centrosymmetric lattice should provide the driving force for the de-trapping of electrons captured in traps under mechanical stimuli. We proposed a possible mechanism to illustrate the unique ML process in Li_2_MgGeO_4_:Mn^2+^; that is, deep traps can replenish electrons to shallow traps when a small number of trapped electrons are released from the shallow traps during mechanical friction, thereby supporting the stable output of ML in the short term. Our results are expected to broaden the horizons of achieving long-term non-decaying ML to eventually break the bottleneck of current short-term non-decaying ML.
